# Tertiary lymphoid structures predict survival and response to neoadjuvant therapy in locally advanced rectal cancer

**DOI:** 10.1038/s41698-024-00533-w

**Published:** 2024-03-02

**Authors:** Qianyu Wang, Wentao Zhong, Xiaofei Shen, Zechen Hao, Meng Wan, Xiaopeng Yang, Ran An, Hongyan Zhu, Huiyun Cai, Tao Li, Yuan Lv, Xing Dong, Gang Chen, Aijun Liu, Junfeng Du

**Affiliations:** 1 Medical Department of General Surgery, The 1st Medical Center, Chinese PLA General Hospital, Beijing, 100853 China; 2 Department of General Surgery, The 7th Medical Center, Chinese PLA General Hospital, Beijing, 100700 China; 3 The Second School of Clinical Medicine, Shanxi Medical University, Taiyuan, 030001 China; 4 The Second School of Clinical Medicine, Southern Medical University, Guangdong, 510515 China; 5 Department of General Surgery, Nanjing Drum Tower Hospital, Affiliated Hospital of Medical School, Nanjing University, Nanjing, 210008 China; 6 Zhongshan School of Medicine, Sun Yat-sen University, Guangzhou, 510030 China; 7 Core Facility for Protein Research, Institute of Biophysics, Chinese Academy of Science, Beijing, 100101 China; 8 Department of Pathology, The 7th Medical Center, Chinese PLA General Hospital, Beijing, 100700 China

**Keywords:** Rectal cancer, Tumour biomarkers, Immunoediting

## Abstract

Tertiary lymphoid structure (TLS) contributes to the anti-tumor immune response, and predicts the prognosis of colorectal cancer patients. However, the potential impact of TLS in shaping the immune status of rectal adenocarcinoma, and the intrinsic relationship between TLS and neoadjuvant therapies (neoTx) remain unclear. We performed hematoxylin-eosin staining, immunohistochemical and biomolecular analyses to investigate TLS and tumor-infiltrating lymphocytes (TILs) in 221 neoTx-treated and 242 treatment-naïve locally advanced rectal cancer (LARC) patients. High TLS density was significantly associated with the absence of vascular invasion, a lower neutrophil-to-lymphocyte ratio, increased TLS maturity, a longer recurrence-free survival (RFS) (hazard ratio [HR] 0.2985 95% confidence interval [CI] 0.1894–0.4706, *p* < 0.0001) and enhanced infiltration of adaptive immune cells. Biomolecular analysis showed that high TLS-score was strongly associated with more infiltration of immune cells and increased activation of immune-related pathways. TLS^+^ tumors in pre-treatment specimens were associated with a higher proportion of good respond (62.5% vs. 29.8%, *p* < 0.0002) and pathological complete remission (pCR) (40.0% vs. 11.1%, *p* < 0.0001), and significantly increased RFS (HR 0.3574 95%CI 0.1489–0.8578 *p* = 0.0213) compared with TLS^-^ tumors in the neoTx cohort, which was confirmed in GSE119409 and GSE150082. Further studies showed that neoTx significantly reduced TLS density and maturity, and abolished the prognostic value of TLS. Our study illustrates that TLS may have a key role in mediating the T-cell-inflamed tumor microenvironment, which also provides a new direction for neoTx, especially neoadjuvant immunotherapy, in LRAC patients.

## Introduction

Rectal cancer (RC) is one of the most common malignancies worldwide^1^. Tumor immune microenvironment (TIME) plays a pivotal role in tumor initiation, progression, and metastasis in several tumors, including colorectal cancer (CRC)^2^. Tumor-infiltrating lymphocytes (TILs), particularly the immunoscore comprised of CD3^+^ and CD8^+^ T cells, emerge as key contributors to the anti-tumor immune response, demonstrating prognostic significance surpassing that of the conventional American Joint Committee on Cancer (AJCC) and Union for International Cancer Control tumor–node–metastasis (TNM) classification system in CRC^3^. Tertiary lymphoid structures (TLSs) are lymphocyte aggregates discovered recently in chronic inflammatory sites, mainly composed of B and T cells, which may support the recruitment of lymphocytes and maintain an immunoresponsive microenvironment^4,5^. Our prior research indicated that tumor-associated TLSs were positively associated with good prognosis in colorectal cancer^6^, while the biological function of TLS in RC remains elusive.

The standard of care for patients with locally advanced rectal cancer (LARC) is neoadjuvant therapies (neoTx) followed by total mesorectal resection^7^. Precisely identifying or effectively promoting the response to neoTx is crucial, as it serves as the fundamental assurance for LARC patients to achieve maximum benefits. Increasing evidence suggests that elevated T-cell infiltration, especially CD8^+^ T cells, in pre-neoTx biopsy tissue of rectal cancer is more likely to be beneficial^8–12^. However, the intrinsic relationship between neoTx and TLS is unknown in LARC.

In this study, we aim to confirm the function of the TLS in conferring local anti-tumor immunity. We additionally explored using TLS as a biomarker to predict sensitivity to neoTx in LARC.

## Results

### The expression and maturity of tumor-associated TLSs in RC

**Fig. 1:**
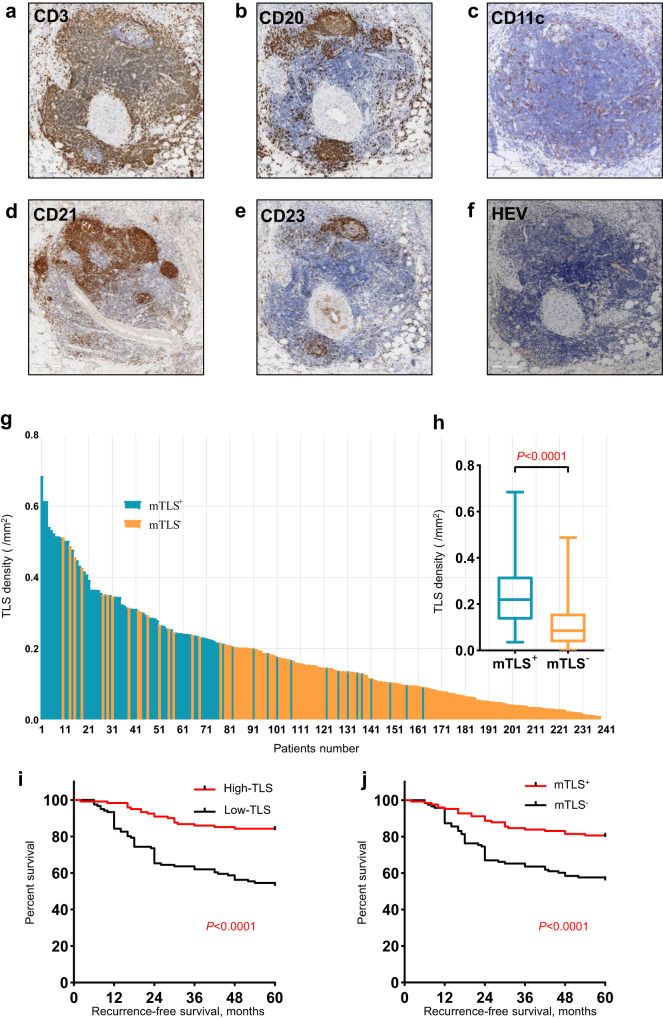
The expression of TLS and its correlates with improved survival in LARC patients. TLSs are defined as dense aggregates of B cells (CD20, **b**) with an adjacent T-cell zone (CD3, **a**) with dendritic cells (DCs, CD11c, **c**) and lacking the surrounding capsule (scale bars, 200 μm). Germinal centers (GCs) were confirmed to contain strictly confined regions of CD21 follicular dendritic cells (**d**) in a field of larger CD23 B cells (**e**) surrounded by PNAd vessels (**f**) (scale bars, 200 μm). **g** Distribution of TLS density and TLS maturation category among 242 rectal cancer patients. **h** The relationship between TLS maturity and TLS density. **i** RFS was compared in patients with high and low TLS density by Kaplan–Meier survival curve. **j** RFS was compared in patients with mTLS^+^ and mTLS^-^ by Kaplan–Meier survival curve. In the graphs, the plotted values encompass the range from the minimum to the maximum, with the inclusion of the median.

Evaluation of the presence and localization of tumor-associated TLSs in LARC patients receiving no treatment (NT) revealed that peritumoral TLS (P-TLS) were identified in 98.3% of all cases—including mature TLS-positive (mTLS^+^) in 52.1% and mature TLS-negative (mTLS^-^) in 47.9% (Fig. 1a–c, Supplementary Fig. 1a–c). Next, we assessed the distribution of TLS density and maturation (Fig. 1d–g). All cases were classified into two groups using the median total TLS density (0.14645 /mm^2^) as the cut-off value, including TLS-high and TLS-low groups. The mTLS^+^ tumor had a significantly higher TLS density compared to the mTLS^-^ tumor (median, 0.2196 vs. 0.0860 /mm^2^, *P* < 0.0001, Fig. 1h). In addition, we also found a high correlation between mTLS% and TLS density (r = 0.621, *P* < 0.001; Supplementary Fig. 1d).

Next, we compared the clinicopathological characteristics of TLS-high and TLS-low groups (Table 1). Compared to the TLS-high group, the TLS-low group was significantly associated with vascular invasion (25.6 vs. 13.2%, *P* = 0.022, Table 1). Considering that the TLS-low group had a higher proportion of pT4 stage (10.7% vs. 3.3%, Table 1), and only 4 patients with pT2 stage, we specifically compared the difference in TLS density between patients with pT3 stage and pT4 stage. TLS density was also statistically significantly lower in patients with pT4 stage than in those with pT3 stage (0.0414 vs. 0.1546 /mm^2^, *P* = 0.0002, Supplementary Fig. 1e). Additionally, the TLS-high group was associated with significantly better systemic inflammation markers, including blood-based neutrophil counts (3.1 vs. 4.0 ×10^9^/L, *P* < 0.001), blood-based lymphocyte counts (2.0 vs. 1.6 ×10^9^/L, *P* < 0.001), blood-based neutrophil-to-lymphocyte ratio (NLR) (1.5 vs. 2.5, *P* < 0.001) compared to the TLS-low group (Table 1). These systemic inflammation markers data suggested that TLS may reduce inflammatory responses and enhance the adaptive immune responses of tumors. TLS density did not show significant associations with other clinical features (Table 1). Additionally, intratumoral TLSs (In-TLSs) were identified in 28.5% of all cases. However, consistent with our previous research^6^, which demonstrated no association of In-TLSs with prognosis, we found no significant correlation between In-TLSs and the prognosis of LARC patients in the NT cohort (Table 2). Therefore, this study primarily focuses on P-TLS.

**Table 1:** Demographics and clinical characteristics between TLS-high and TLS-low group in LARC patients

Characteristics	TLS-high (*n* = 121)	TLS-low (*n* = 121)	*P* value
Sex			0.156
Male	91 (75.2)	81 (62.0)	
Female	30 (24.8)	40 (38.0)	
Age (years)	57 (49–64)	60 (52–64)	0.151
pT			0.111
2	1 (0.8)	3 (2.5)	
3	116 (95.9)	105 (86.8)	
4	4 (3.3)	13 (10.7)	
pN			0.282
0	73 (60.3)	65 (53.7)	
1	38 (31.4)	43 (35.5)	
2	10 (8.3)	13 (10.7)	
pStage			0.363
II	73 (60.3)	65 (53.7)	
III	48 (39.7)	56 (46.3)	
Tumor location			0.431
Low	52 (43.0)	45 (37.2)	
Medium/high	69 (57.0)	76 (62.8)	
Perineural invasion			0.868
positive	21 (17.4)	23 (19.0)	
negative	100 (82.6)	98 (81.0)	
Vascular invasion			0.022
positive	16 (13.2)	31 (25.6)	
negative	105 (86.8)	90 (74.4)	
Tumor grade			0.410
G1/2	116 (95.9)	112 (92.6)	
G3	5 (4.1)	9 (7.4)	
mTLS stage			<0.001
mTLS^+^	87 (71.9)	37 (30.6)	
mTLS^-^	34 (28.1)	84 (69.4)	
Neutrophil (×10^9^/L)^a^	3.1 (2.4–3.8)	4.0 (3.2–4.9)	<0.001
Lymphocyte (×10^9^/L)^a^	2.0 (1.7–2.5)	1.6 (1.2–1.9)	<0.001
NLR^a^	1.5 (1.3–1.8)	2.5 (2.1–2.9)	<0.001
Adjuvant chemotherapy			0.520
FL/Capecitabine	65 (53.7)	59 (48.8)	
FOLFOX/XELOX	56 (46.3)	62 (51.2)	

*LARC* locally advanced rectal cancer, *TLS* tertiary lymphoid structure, *mTLS stage* mature TLS stage, *G1/2* Moderate or poor, *G3* well, *NLR* neutrophil-to-lymphocyte ratio, *FL* 5-fluorouracil + leucovorin, *FOLFOX* 5-fluorouracil + leucovorin + oxaliplatin, *XELOX* capecitabine + oxaliplatin; ^a^blood-based.

**Table 2:** Cox proportional hazards regression models for the predictors of RFS in the LARC cohort (*n* = 242)

Variables	Univariate Analysis		Multivariate Analysis			
RFS	HR (95% CI)	*P* value	Model A		Model B	
			HR (95% CI)	*P* value	HR (95% CI)	*P* value
Sex (male vs female)	1.544 (0.967–2.466)	0.069				
Age >60 vs. ≤60	1.005 (0.641–1.576)	0.983				
Tumor grade (G1/2 vs. G3)	1.257 (0.508–3.112)	0.621				
Tumor location (Medium/high vs. low)	0.904 (0.574–1.422)	0.662				
In-TLS (no vs. yes)	1.504 (0.877–2.577)	0.138				
Perineural invasion (no vs. yes)	0.478 (0.292–0.785)	0.004	0.751 (0.428–1.315)	0.316	0.721 (0.407–1.274)	0.260
Vascular invasion (no vs. yes)	0.354 (0.222–0.567)	<0.001	0.771 (0.446–1.331)	0.350	0.791 (0.449–1.393)	0.417
TNM stage (III vs. II)	3.498 (2.157–5.673)	<0.001	3.496 (2.108–5.799)	<0.001	3.017 (1.822–4.997)	<0.001
CD8^+^ T cells (low vs. high)	3.718 (2.212–6.250)	<0.001	1.935 (1.069–3.502)	0.029	2.640 (1.517–4.593)	0.001
TLS density (low vs. high)	3.513 (2.109–5.851)	<0.001	2.895 (1.612–5.200)	<0.001		
mTLS stage (mTLS- vs mTLS + )	2.567 (1.592–4.139)	<0.001			1.885 (1.125–3.159)	0.016
Adjuvant chemotherapy (FL/Capecitabine vs FOLFOX/XELOX)	0.839 (0.571–1.398)	0.621				

### TLSs are robust predictors of RFS

To validate the prognostic value of TLS in LARC, we evaluated the density and maturity of TLS in 242 treatment-naïve LARC surgical specimens from two institutes. Kaplan–Meier analysis showed that the TLS-high group exhibited better RFS compared to the TLS-low group (hazard ratio [HR] 0.2985 95% CI 0.1894–0.4706, *P* < 0.0001, Fig. 1i). In addition, compared to mTLS^-^ tumor, mTLS^+^ tumor also has better RFS (HR 0.3899 95% CI 0.2473–0.6145, *P* < 0.0001, Fig. 1j). Moreover, univariate Cox regression analysis revealed that RFS was significantly associated with pTNM stage, CD8^+^ T cells density, perineural invasion, vascular invasion and TLS density (or TLS maturation) (Table 2). Multivariate Cox regression analysis showed that TLS density (or TLS maturation), CD8 + T cells density and pTNM stage were independent predictors of RFS (Table 2). These findings suggested that the TLS may play a significant role in suppressing tumor progression.

### TLSs mediate characteristics of the T-cell-inflamed tumor microenvironment

**Fig. 2:**
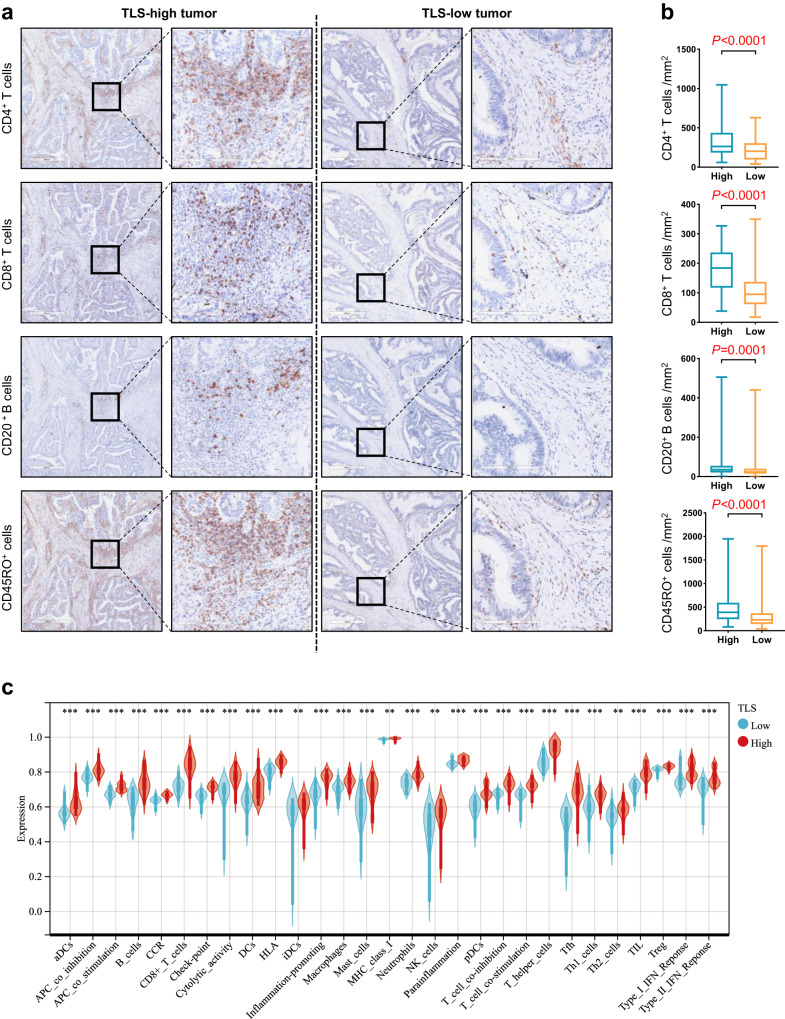
The role of TLSs in the TIME. **a** TIL expression in primary resected rectal tissue was categorized into NT patients with low and high TLS density (scale bars, 600 μm and 200 μm). **b** CD4^+^ T cells, CD8^+^ T cells, CD20^+^ B cells and CD45RO^+^ cells were compared in NT patients with high and low TLS density. **c** Gene set enrichment analysis with hallmark gene sets in the NT cohort (30 high vs. 30 low TLS density). *P < 0.05, **P < 0.01, ***P < 0.001, ****P < 0.0001. In the graphs, the plotted values encompass the range from the minimum to the maximum, with the inclusion of the median.

As adaptive immune cells infiltrating RC tumors may originate from TLS or secondary lymphoid organs (SLO), we first hypothesized that TLS could enhance the local proliferation and infiltration of adaptive immune cells into tumors. To test this hypothesis, we performed immunohistochemistry (IHC) to quantify the density of CD4^+^ T cells, CD8^+^ T cells, CD20^+^ B cells and CD45RO^+^ cells on 242 LARC tumor tissues from the NT cohort (Fig. 2a). Our findings revealed that CD4^+^ T cells (median, 263.5 vs. 201.6 /mm^2^, *P* < 0.0001), CD8^+^ T cells (median, 184.2 vs. 94.86 /mm^2^, *P* < 0.0001), CD20^+^ B cells (median, 35.32 vs. 23.07 /mm^2^, *P* = 0.0001) and CD45RO^+^ cells (median, 389.7 vs. 231.6 /mm^2^, *P* < 0.0001) were significantly enriched in the TLS-high compared with the TLS-low group (Fig. 2b).

To further understand the biological importance of TLSs in LARC patients, thirty TLS-low and thirty TLS-high specimens were obtained from respective groups and analyzed by RNA sequencing. The RNA sequencing data showed that TLS-high samples exhibited a higher enrichment of 21 TLS-score compared to TLS-low samples (Supplementary Fig. 1f). Moreover, the RNA sequencing data confirmed that TLS-high tumors contained a greater abundance of immune cells (B cells, CD8^+^ T cells, CD4^+^ T cells, T helper 1 cells, T follicular helper cells, T regulatory cells, TILs and macrophages) and increased activation of immune-related pathways (antigen presentation pathway, interferon pathway and immune-potentiating chemokines) compared with TLS-low tumors (Fig. 2c). These results suggest that TLSs may have a key role in mediating T-cell-inflamed tumor microenvironment, and thus, potentially contributing to the optimal anti-tumor immune response.

### TLSs in pre-treatment specimens promote neoTx response in LARC patients

**Fig. 3:**
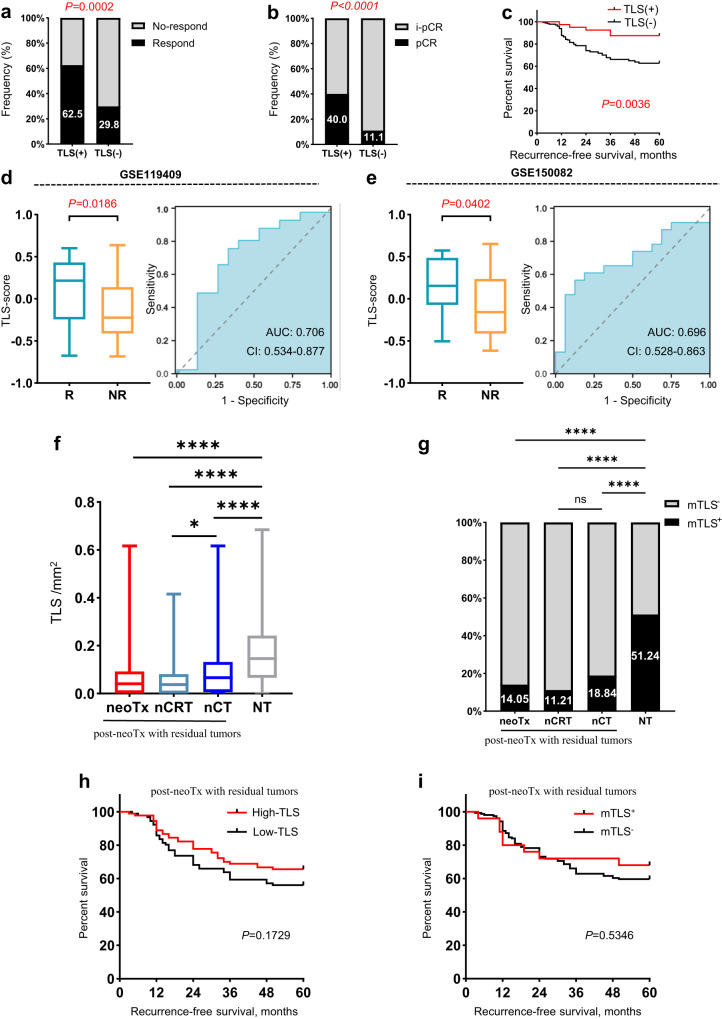
The interrelationship between TLS and neoTx. **a, b** The expression ratio of TLS in pre-neoTx specimens was compared in responders and non-responders, pCR and ipCR. Analysis was performed by Fisher’s exact test. **c** RFS was compared in patients with TLS^+^ and TLS^−^ tumors by Kaplan–Meier survival curve. **d, e** 21 TLS-scores were compared in responders and non-responders from GSE119409 (*n* = 56, *P* = 0.0186) and GSE150082 (*n* = 39, *P* = 0.0402). R, responders; NR, non-responders. TLS density (**f**) and TLS maturity (**g**) were compared in post-neoTx with residual tumors and NT patients. RFS was compared by the Kaplan–Meier survival curve in patients with high and low TLS density (**h**) and mTLS^+^ and mTLS^−^ (**i**) in the post-neoTx with residual tumors cohort. *P < 0.05, **P < 0.01, ***P < 0.001, ****P < 0.0001. In the graphs, the plotted values encompass the range from the minimum to the maximum, with the inclusion of the median.

To further assess the role of TLS in predicting sensitivity to neoTx, we enrolled 221 pre-neoTx biopsy specimens in neoTx-treated RC patients. In contrast to the expression of TLS in surgical resection specimens from the NT cohort (98.3%), TLS was detected in pre-neoTx biopsy tissues of only 18.1% of patients. This discrepancy underscores the difference between small biopsy specimens and surgical resection. Accordingly, patients were categorized into TLS^+^ and TLS^−^ groups based on the expression of TLS in pre-neoTx biopsy tissues. The baseline characteristics of patients in these two groups are summarized in Supplementary Table 1. In the TLS^+^ group, the frequency of responders was significantly higher than that in the TLS^-^ group (62.5% vs. 29.8%, *P* = 0.0002, Fig. 3a). Furthermore, there were also more patients achieving pCR in the TLS^+^ group compared to the TLS^-^ group (40.0% vs. 11.1%, *P* < 0.0001, Fig. 3b). Subsequent univariate and multivariate logistic regression analysis demonstrated that patients with TLS on pre-neoTx biopsies were associated with a better response to neoTx (univariate odds ratio 3.920, 95% CI 1.918–8.012, *p* < 0.001, Supplementary Table 2). Multivariate logistic regression analysis suggests that CD8^+^ T cells, TLS and neoadjuvant therapy regimens are independent predictors of patient sensitivity to neoadjuvant therapy (Supplementary Table 2). All these results indicated that TLS may favor patients’ prognosis. Supporting this conclusion, we identified that TLS^+^ patients experienced better RFS compared to the TLS- group (HR 0.4233, 95% CI 0.2373–0.7551, *P* = 0.0036, Fig. 3c).

To further examine the predictive values of TLS in neoTx-treated LARC patients, we verified the predictive effect of the 21 TLS-hallmark genes using GSE119409 (n = 56) and GSE150082 (n = 39). We found that responders exhibited a significantly higher 21 TLS-score than non-responders in the GSE119409 (median, 0.2165 vs. −0.2232, *P* = 0.0186) and GSE150082 (median, 0.1547 vs. −0.1574, *P* = 0.0402) dataset (Fig. 3d, e). Receiver operating characteristic curve analysis demonstrated that TLS-score accurately predicted the response to neoTx, with a high area under the curve (AUC) in the GSE119409 (0.706; 95% CI, 0.534–0.877) and GSE150082 (0.696; 95% CI, 0.528–0.863) datasets (Fig. 3d, e). These results suggest that TLS is a critical factor in promoting the efficacy of neoTx.

### neoTx reduces TLS density and maturity, and abolishes the prognostic value of TLS

To evaluate the effect of neoTx on TLS, we compared the TLS density and maturation between 185 post-neoTx patients with residual tumors and 242 NT patients. We found that the TLS density and mTLS^+^ were significantly lower in neoTx-treated patients with residual tumors compared with NT patients (TLS density: median, 0.0413 vs 0.1465 /mm^2^, *P* < 0.0001, Fig. 3f; mTLS^+^: 14.05 vs. 51.24%, *P* < 0.0001, Fig. 3g).

Afterward, we categorized patients treated with neoTx into two groups: those who underwent neoadjuvant chemoradiotherapy (nCRT) and those who underwent neoadjuvant chemotherapy (nCT). Subsequent subgroup analysis revealed a statistically significant reduction in TLS density among nCRT patients compared to nCT and NT patients (0.0374 vs. 0.0663 /mm^2^, *P* = 0.0239 vs. nCT; 0.0374 vs. 0.1465 /mm^2^, *P* < 0.0001 vs. NT; Fig. 3f). Additionally, mTLS^+^ was markedly higher in NT patients than in nCRT and nCT patients (51.24 vs. 11.21%, *P* < 0.0001 vs. nCRT; 51.24 vs. 18.84%, *P* < 0.0001 vs. nCT; Fig. 3g). No statistically significant difference in mTLS^+^ was observed between nCRT and nCT patients (Fig. 3g). Surprisingly, TLS density and mTLS^+^ lost prognostic value in the neoTx-treated incomplete pathological remission (ipCR) patients (Fig. 3h, i).

## Discussion

Over the past decade, TIME has been extensively studied due to remarkable advances in immunotherapy^13^. TLSs are lymphocyte aggregates found in nonlymphoid tissues and sharing characteristics and functions similar to SLO^4,5^. TLSs are predictors of positive prognosis in CRC^6,14,15^, while the biological function of TLS in RC remains poorly understood. Many studies often confuse colon and rectal cancer^6,14,15^, however, these distinct entities—rectal, left and right colon cancer—may exhibit varying characteristics and functions within TIME^16–18^. Therefore, this study focused on the role of TLS in the TIME of rectal cancer.

In this study, we quantitatively and objectively assessed TLS density and maturity in treatment-naïve LARC surgical specimens from two institutes. Our study showed that the presence of TLS was observed in 98.3% of LARC patients, which was roughly consistent with other CRC studies^6,14^. We then found that the TLS-high group had better RFS compared to the TLS-low group in the LARC cohort.

TLSs may serve as mediators of anti-tumor immune response through TILs. A growing body of research suggests that immune infiltration in TLS-high tumors tends to be biased towards a T helper 1 or cytotoxic effector state^19^ and exhibits the expression of a series of immune checkpoint molecules^20^. In our study, we discovered a significant association between TLS-high tumors and a T-cell inflammatory phenotype, as evidenced by IHC and RNA sequencing. Specifically, we found that high TLS density correlated with the heightened infiltration of adaptive immune cells, such as CD4^+^ T cells, CD8^+^ T cells, CD20^+^ B cells and CD45RO^+^ cells. To further explore the intricate relationship between TLSs and TIME, we conducted RNA sequencing on 30 TLS-high and 30 TLS-low tumors. Consistent with our initial findings, the RNA sequencing data show that TLSs can promote the infiltration of adaptive immune cells and the activation of immune-related pathways. These results strongly suggest that TLSs may play a pivotal role in enhancing tumor-specific immune responses.

In our subgroup analysis, our study showed that TLS-low was correlated with multiple factors that promote tumor progression, such as vascular invasion. Additionally, an interesting observation emerged, indicating that patients classified as pT4 stage exhibited lower TLS density compared to those with pT3 stage tumors. One conceivable explanation for this phenomenon is that tumor-associated TLS might exert an inhibitory effect on tumor growth. Firstly, the specific localization of TLS may increase the chances of encounters between tumor-associated antigens and rare, matching lymphocytes. This, in turn, could potentially facilitate the induction of more robust and broader immune responses^4^. Furthermore, the local priming of T and B cell pools within TLSs might result in expedited immune responses by circumventing the need for DCs and lymphocytes to travel to and from SLO^4,21^. The secretion of survival factors by TLS-associated fibroblasts and other cell subsets supports lymphocyte homeostasis within TLSs^22,23^, suggesting a possible contribution to the sustained presence of tumor-reactive T cells at tumor sites. Collectively, these factors may act synergistically to inhibit tumor growth.

The NLR reflects the equilibrium between the two facets of the immune system: acute and chronic inflammation (neutrophil count) and adaptive immunity (lymphocyte count)^24^. An elevated NLR is indicative of a systemic inflammatory response, and is a poor prognostic marker in multiple tumor types, including CRC^25,26^. Our data show that TLS-high is associated with lower NLR, implying that TLS can promote anti-tumour immune response and reduce inflammation. However, it remains uncertain whether an elevated NLR might impede the development of TLS.

There is growing evidence that TLSs are significantly associated with the benefit of immune checkpoint inhibitors (ICIs)^27–30^. However, it remains an open question whether the predictive value of TLS is exclusive to ICIs or extends to predicting sensitivity to neoTx, particularly in LARC patients. In breast cancer, four studies have demonstrated a significant association between TLS and the efficacy of neoadjuvant chemotherapy^31–34^. In the NT cohort, TLS was observed in 98.3% of cases. In stark contrast, only 18.1% of pre-neoTx biopsy specimens from the neoTx cohort exhibited TLS. This discrepancy may be attributed to the limited area of pre-neoTx endoscopic biopsies, potentially resulting in numerous false negatives. Nonetheless, our data still suggests that TLS^+^ group exhibited a better treatment response and a higher proportion of pCR compared to the TLS^-^ group. Furthermore, we observed that responders had higher 21 TLS-scores compared to non-responders in the GSE119409 and GSE150082. In non-small cell lung cancer, however, two studies have showed that TLS could predict the sensitivity to ICIs but not to chemotherapy^27,35^. A potential explanation for this discrepancy is that chemotherapy may impair TLS maturation^36^. The mechanisms by which TLSs contribute to the response to neoTx remain to be clarified and require further research.

It is widely acknowledged that neoTx can reshape and enhance the TIME, and rectal cancer is no exception^37,38^. While numerous studies have delved into the impact of immunotherapy on TLSs^39–42^, few have investigated the impact of neoTx on TLSs. In a study on lung cancer, it was discovered that neoadjuvant chemotherapy compromised the prognostic value of TLS density, possibly linked to impaired formation of germinal centers within TLSs^36^. Additionally, Väyrynen et al. ^43^ demonstrated that neoTx significantly reduced TLS density in colorectal cancer patients. In this context, our study presents evidence of the impact of neoTx on tumor-associated TLS and its prognostic implications in rectal cancer. Our findings revealed that neoTx reduced TLS and eliminated its prognostic value. Subgroup analysis further indicated that mTLS^+^ and TLS density were statistically significantly higher in NT patients than in those undergoing nCRT and nCT. TLS density, but not mTLS, was statistically significantly lower in nCRT patients than in nCT patients. Given the impact of neoTx on the formation of TLS, further research is necessary to explore the contradictions between neoTx and TLS.

Our study had certain limitations. First, being a retrospective study, there is a potential for selection bias. Secondly, the biopsies exhibited high heterogeneity and may not have been entirely representative of the entire tumor. Consequently, false-negative TLS or TLS scores could be present in the pre-neoTx biopsy. Lastly, TLS may interact differently with various anti-tumor drugs. However, owing to the limited sample size, we categorized these patients into two groups: nCRT and nCT, without specific subgrouping based on distinct anti-tumor drugs. Given these considerations, our forthcoming research will concentrate on investigating the correlation between TLS and various anti-tumor drugs.

Our study represents the systematic exploration of the relationship between TLSs and the TIME in LARC patients. Furthermore, we observed that TLSs could enhance prognostic outcomes and improve the response to neoTx. However, it is noteworthy that our findings also indicated that neoTx diminishes TLS and eliminates its prognostic significance. In conclusion, our study suggests that TLS induction correlates with favorable anti-tumor immune responses, thus potentially serving as an indicative factor for immunotherapy in LARC.

## Methods

### Patient data

This study included 242 LARC patients receiving no treatment (NT) at two different institutes, including the 7th Medical Center of Chinese PLA General Hospital and the Affiliated Drum Tower Hospital of Nanjing University Medical School between January 2015 to January 2017. All patients underwent curative surgical resection without neoTx, such as chemotherapy (CT) or chemoradiotherapy (CRT). Additionally, this study included a different cohort of 221 neoTx-treated RC patients at the two institutions from January 2013 to January 2018. The pre-neoTx endoscopic biopsies were obtained through transenteroscopic biopsy in neoTx-treated RC patients. Furthermore, 185 residual cancer tissue samples after neoTx were obtained in this neoTx-treated RC patients. All patients underwent routine follow-up for 5 years, and RFS was defined as the interval between surgical treatment and disease recurrence. This study is compliant with the ‘Guidance of the Ministry of Science and Technology (MOST) for the Review and Approval of Human Genetic Resources’. Sampling and handling of any patient material was performed in accordance with the ethical principles of the Declaration of Helsinki. This includes written informed consent from all donors and the approval by the Institute Research Ethics Committees of the 7th Medical Center of Chinese PLA General Hospital (2020–32) and Affiliated Drum Tower Hospital of Nanjing University Medical School (2019-312-01).

### Clinicopathological characteristics

Clinicopathological features included age, sex, tumor location (tumor distance from the anal verge), adjuvant chemotherapy, tumor grade, tumor stage, vascular invasion and perineural invasion. As systemic inflammation markers, the blood-based NLR was calculated based on pre-operative data. Blood-based NLR = blood-based total neutrophil count (10^9^/L)/ blood-based total lymphocyte count (10^9^/L). Following previously established procedures^6^, we meticulously reviewed all hematoxylin and eosin-stained sections, selected representative sections, and cut sections into 4-μm-thick slices. The MoticEasyScan Infinity was used to scan all hematoxylin and eosin-stained sections, and Motic DSAssistant was used to analyze.

### Assessment of tumour regression score

Tumor regression score (TRS) in the resected specimen post-neoTx was calculated using the recommendations provided by the College of American Pathologists and AJCC guidelines^44^. The TRS is categorized as follows: 0, no viable cancer cells; 1, single cells or rare small groups of cancer cells; 2, residual cancer outgrown by fibrosis; and 3, extensive residual cancer with no evident tumor regression^44^. TRS was independently determined by two pathologists (RA and HZ) who were unaware of the patient’s characteristics and results. The difference between the two pathologists’ conclusions was arbitrated by the third pathologist (AL). Patients with TRS 0 and 1 were considered responders, while those with TRS 2 and 3 were categorized as non-responders. TRS 0 was considered pCR and TRS 1–3 was considered incomplete pathological remission (ipCR).

### Immunohistochemistry

Immunohistochemistry (IHC) was performed on 4-μm-thick sections, as previously described^6^. The slides were deparaffinized, rehydrated, incubated with 3% H_2_O_2_, subjected to heat-mediated antigen repair, incubated with primary and secondary antibodies, incubated with diaminobenzidine, followed by hematoxylin staining, and finally dehydrated and sealed in neutral resin. Supplementary Table 3 shows details of the primary and secondary antibodies.

### Assessment of TLSs and TILs

TLSs are defined as dense aggregates of B cells (CD20) with an adjacent T-cell zone (CD3) accompanied by dendritic cells (DCs, CD11c) and lacking the surrounding capsule (Fig. 1a–c), in accordance with previous descriptions^6^. The germinal center (GC) was confirmed to contain strictly confined regions of CD21+ follicular dendritic cells (FDC) in a field of larger CD23 + B cells surrounded by PNAd+ high endothelial venules (HEV) (Fig. 1d–f). TLS was divided into peritumoral TLS (P-TLS) and intratumoral TLS (In-TLS) based on their location to tumor invasive margins (Supplementary Fig. 1a–c). The number of TLSs per square millimeter in a 7-mm area around the tumor was defined as the P-TLS density, and the number of TLSs per square millimeter in the tumor regions was defined as the In-TLS density^6,14^. Patients were divided into high and low TLS density groups by the median of TLS density. Patients with GC-positive TLSs were defined as the mature TLS-positive (mTLS^+^) group, while the remaining patients were defined as the mature TLS-negative (mTLS^−^) group. The numbers of mTLS were counted and the proportion of mTLS in all TLSs within each patient was calculated as the mTLS%.

IHC was performed on successive sections to further assess TILs, such as CD4^+^ T cells, CD8^+^ T cells, CD20^+^ B cells and CD45RO^+^ cells. For each patient, we calculated the immune cell density in the tumor area. The sections were scanned using Leica CS2 and analyzed with Aperio ImageScope. Each section was subject to quality control and digitally annotated (VHK, DM and JH) using the Halo imaging analysis software (Indica Labs, Corrales, NM, USA) from Core Facility for Protein Research, Institute of Biophysics, Chinese Academy of Science.

### RNA-seq and analysis

We performed RNA-seq analysis on 60 patients (30 TLS-high and 30 TLS-low samples) fresh-frozen tissues. Total RNA was extracted from the tissue using TRIzol® Reagent according the manufacturer’s instructions (Invitrogen) and genomic DNA was removed using DNase I (TaKara). RNA degradation and contamination was monitored on 1% agarose gels. Then RNA quality was determined by 2100 Bioanalyser (Agilent Technologies) and quantified using the ND-2000 (NanoDrop Technologies). Only high-quality RNA sample (OD260/280 = 1.8 ~ 2.2, OD260/230 ≥ 2.0, RIN ≥ 8.0, 28 S:18 S ≥ 1.0, >1 μg) was used to construct sequencing library. RNA purification, reverse transcription, library construction and sequencing were performed at Shanghai Majorbio Bio-pharm Biotechnology Co., Ltd. (Shanghai, China) according to the manufacturer’s instructions (Illumina, San Diego, CA). The transcriptome library was prepared following TruSeqTM RNA sample preparation Kit from Illumina (San Diego, CA) using 1 μg of total RNA. Shortly, messenger RNA was isolated according to polyA selection method by oligo(dT) beads and then fragmented by fragmentation buffer first. Secondly, double-stranded cDNA was synthesized using a SuperScript double-stranded cDNA synthesis kit (Invitrogen, CA) with random hexamer primers (Illumina). Then the synthesized cDNA was subjected to end-repair, phosphorylation and ‘A’ base addition according to Illumina’s library construction protocol. Libraries were size selected for cDNA target fragments of 300 bp on 2% Low Range Ultra Agarose followed by PCR amplified using Phusion DNA polymerase (NEB) for 15 PCR cycles. After quantified by TBS380, paired-end RNA-seq sequencing library was sequenced with the Illumina NovaSeq 6000 sequencer (2 × 150 bp read length).

### Assessment of TLS score

Twenty-one TLS-hallmark genes (*CCL2, CCL3, CCL4, CCL5, CCL8, CCL18, CCL19, CCL21, CXCL9, CXCL10, CXCL11, CXCL13, CD79B, CD1D, CCR6, LAT, SKAP1, CETP, EIF1AY, RBP5 and PTGDS*) obtained from two published datasets^29,45^ were used as TLS scores. The single-sample gene set enrichment analysis (ssGSEA) and gene set variation analysis (GSVA) R package were used to calculate the TLS score in R (version 4.2.1).

### Gene expression databases

The rectal cancer gene expression profiles used in this study were downloaded from the publicly available GEO (http://www.ncbi.nlm.nih.gov/geo/) databases. We included LARC patients receiving neoTx from the GSE119409 (*n* = 66) and GSE150082 (*n* = 39) datasets, and obtained the gene expression profiles in pre-neoTx specimens and response to neoTx. 10/66 patients had no sensitivity in the GSE119409 dataset. GSE119409 (*n* = 56) and GSE150082 (*n* = 39) datasets were used to evaluate the role of TLS score in predicting sensitivity to neoadjuvant therapy.

### Analysis of immunological characteristics

We used the GSVA package in ssGSEA to determine the immunological characteristics, such as immune cells and immune-related pathways^46^. The detailed information on the gene enrichment of individual immune cells and immune-related pathways in Supplementary Table 4.

### Statistical analysis

The statistical analysis software used in this study was R version 4.2.1, SPSS version 25 and GraphPad Prism version 8. Fisher’s exact test and Mann–Whitney U test were used to calculate *P* values. RFS and independent risk factors for prognosis were analyzed by Kaplan–Meier and log-rank survival analyses, and univariate and multivariate analyses. All statistics were examined using two-sided analysis, with *P* < 0.05 regarded as statistically significant.

### Reporting summary

Further information on research design is available in the Nature Research Reporting Summary linked to this article.

### Supplementary information

Supplementary information

REPORTING SUMMARY

## Data Availability

The datasets used and/or analyzed in the current study presented in the study are included in the article/ Supplementary information. Further inquiries can be directed to the corresponding author (M.D. Junfeng Du, E-mail: dujunfeng@301hospital.com.cn) on reasonable request. The ethics committee and informed consent signed by the participants does not allow for the de-identified RNA sequencing data to be deposited into a secure access-controlled repository.

## Code availability

All scripts implemented in R version 4.2.1 are available from in the Supplementary information.
