# Supplementary material for: Tertiary lymphoid structures predict survival and response to neoadjuvant therapy in locally advanced rectal cancer
Source: NPJ Precis Oncol. 2024 Mar 2;8:61. doi: 10.1038/s41698-024-00533-w (PMC10908779; doi:10.1038/s41698-024-00533-w)
Supplement: Supplementary file 1 — Supplementary information [file 41698_2024_533_MOESM1_ESM.pdf]

Supplementary information

1. Supplementary Tables

2. Supplementary Figures

3. Supplementary Code

Supplement Table 1 Relationship between TLS and clinicopathological characteristics in pre-neoTx biopsy tissues

	**pre-neoTx biopsy tissues (n=221)**		
**Characteristics**	**TLS$^+$ (n=40)**	**TLS$^-$ (n=181)**	**P value**
**Sex**	**n = 40 (%)**	**n = 181 (%)**	0.841
Male	31 (77.5)	135 (74.6)	
Female	9 (22.5)	46 (25.4)	
**Age(years)**	58 (52-62)	58 (50-64)	0.977
**Biopsy specimens**	**n = 40 (%)**	**n = 181 (%)**	0.829
1	1 (2.5)	1 (0.6)	
2	3 (7.5)	16 (8.8)	
3	18 (45.0)	89 (49.2)	
4	17 (42.5)	68 (37.6)	
5	1 (2.5)	7 (3.9)	
**Area of biopsy (mm^2)**	9.049 (7.663-10.48)	8.654 (7.994-9.655)	0.478
**(y)pT (%)**	**n = 40 (%)**	**n = 181 (%)**	**<0.001**
(y)pT0	16 (40.0)	20 (11.0)	
(y)pT1	0 (0.0)	1 (0.6)	
(y)pT2	10 (25.0)	30 (16.6)	
(y)pT3	12 (30.0)	119 (65.7)	
(y)pT4	2 (5.0)	11 (6.1)	
**(y)pN (%)**	**n = 40 (%)**	**n = 181 (%)**	**0.001**
(y)pN0	34 (85.0)	106 (58.6)	
(y)pN1	6 (15.0)	56 (30.9)	
(y)pN2	0 (0.0)	19 (10.5)	
**(y)pM (%)**	**n = 40 (%)**	**n = 181 (%)**	>0.9999
(y)pM0	40 (100.0)	181 (100.0)	
(y)pM1	0 (0.0)	0 (0.0)	
**(y)Stage (%)**	**n = 40 (%)**	**n = 181 (%)**	**<0.001**
0	16 (40.0)	20 (11.0)	
1	9 (22.5)	32 (17.7)	
2	7 (17.5)	57 (31.5)	
3	8 (20.0)	72 (39.8)	
**pCR**	**n = 40 (%)**	**n = 181 (%)**	**<0.001**
Yes	16 (44.4)	20 (13.0)	
No	24 (55.6)	161 (87.0)	
**Respond**	**n = 40 (%)**	**n = 181 (%)**	**<0.001**

Yes	25 (62.5)	54 (29.8)	
No	15 (37.5)	127 (70.2)	
**Perineural invasion**	**n = 24 (%)**	**n = 161 (%)**	>0.9999
Yes	4 (16.7)	29 (18.0)	
No	20 (83.3)	132 (82.0)	
**Vascular invasion**	**n = 24 (%)**	**n = 161 (%)**	>0.9999
Yes	4 (16.7)	28 (17.4)	
No	20 (83.3)	133 (82.6)	
**Tumor grade**	**n = 24 (%)**	**n = 161 (%)**	0.2590
Moderate/poor	22 (91.7)	129 (80.1)	
Well	2 (8.3)	32 (19.9)	

neoTx, neoadjuvant therapies; pCR, pathological complete remission; TLS, tertiary lymphoid structure.

Supplementary Table 2 Cox proportional hazards regression models for the predictors of respond to neoTx in the neoTx-treated cohort (n=221)

**Variables**	**Univariate Analysis**		**Multivariate Analysis**	
	**HR (95% CI)**	**P value**	**HR (95% CI)**	**P value**
Sex (male vs. female)	0.784 (0.419-1.469)	0.448		
Age >60 vs. ≤60	1.307 (0.747-2.287)	0.348		
Tumor grade (G3 vs. G1/2)	1.349 (0.694-2.622)	0.378		
CD8+ T cells in biopsy (High vs. Low)	8.624 (4.448-16.720)	**<0.001**	8.226 (4.068-16.632)	**<0.001**
TLS density in biopsy (yes vs. no)	3.920 (1.918-8.012)	**<0.001**	4.915 (2.085-11.585)	**<0.001**
Neoadjuvant therapy type (Chemoradiotherapy vs. chemotherapy)	2.881 (1.516-5.474)	**0.001**	2.642 (1.251-5.579)	**0.011**

TLS, tertiary lymphoid structure; PFS, progression-free survival; HR, hazard ratio; CI, confidence interval; G1/2, Moderate or poor; G3, well.

Supplementary Table 3 Antibodies

Antibody	Species	Dilution	Source	Identifier
Anti-CD3	Mouse	1:150	ZSGB-BIO	TA506064
Anti-CD4	Rabbit	1:200	Abcam	ab133616
Anti-CD8	Rabbit	1:300	Abcam	ab101500
Anti-CD20	Mouse	1:100	Abcam	ab9475
Anti-CD21	Mouse	1:100	ZSGB-BIO	TA327627
Anti-CD23	Mouse	1:150	ZSGB-BIO	TA801554
Anti-CD45RO	Mouse	1:1000	Abcam	ab23
Anti-CD68	Rabbit	1:4000	Abcam	ab213363
Anti-PNAd	Rat	1:100	Biolegend	120802

Supplementary Table 4 Gene enrichment analysis for individual immune cells and immune-related pathways	
aDCs	CD83;LAMP3;CCL1
APC_co_inhibition	C10orf54;CD274;LGALS9;PDCD1LG2;PVRL3
APC_co_stimulation	CD40;CD58;CD70;ICOSLG;SLAMF1;TNFSF14;TNFSF15;TNFSF18;TNFSF4;TNFSF8;TNFSF9
B_cells	BACH2;BANK1;BLK;BTLA;CD79A;CD79B;FCRL1;FCRL3;HVCN1;RALGPS2
CCR	CCL16;TPO;TGFBR2;CXCL2;CCL14;TGFBR3;IL11RA;CCL11;IL4I1;IL33;CXCL12;CXCL10;BMPER;BMP8A;CXCL11;IL21R;IL17B;TNFRSF9;ILF2;CX3CR1;CCR8;TNFSF12;CSF3;TNFSF4
CD8+_T_cells	CD8A
Check-point	IDO1;LAG3;CTLA4;TNFRSF9;ICOS;CD80;PDCD1LG2;TIGIT;CD70;TNFSF9;ICOSLG;KIR3DL1;CD86;PDCD1;LAIR1;TNFRSF8;TNFSF15;TNFRSF14;IDO2;CD276;CD40;TNFRSF4;TNFSF14;HHLA2
Cytolytic_activity	PRF1;GZMA
DCs	CCL17;CCL22;CD209;CCL13
HLA	HLA-E;HLA-DPB2;HLA-C;HLA-J;HLA-DQB1;HLA-DQB2;HLA-DQA2;HLA-DQA1;HLA-A;HLA-DMA;HLA-DOB;HLA-DRB1;HLA-H;HLA-B;HLA-DRB5;HLA-DOA;HLA-DPB1;HLA-DRA;HLA-DRB6;HLA-L;HLA-F;HLA-G;HLA-DMB;HLA-DPA1
iDCs	CD1A;CD1E
Inflammation-promoting	CCL5;CD19;CD8B;CXCL10;CXCL13;CXCL9;GNLY;GZMB;IFNG;IL12A;IL12B;IRF1;PRF1;STAT1;TBX21
Macrophages	C11orf45;CD68;CLEC5A;CYBB;FUCA1;GPNMB;HS3ST2;LGMN;MMP9;TM4SF19
Mast_cells	CMA1;MS4A2;TPSAB1
MHC_class_I	B2M;HLA-A;TAP1
Neutrophils	EVI2B;HSD17B11;KDM6B;MEGF9;MNDA;NLRP12;PADI4;SELL;TRANK1;VNN3
NK_cells	KLRC1;KLRF1
Parainflammation	CXCL10;PLAT;CCND1;LGMN;PLAUR;AIM2;MMP7;ICAM1;MX2;CXCL9;ANXA1;TLR2;PLA2G2D;ITGA2;MX1;HMOX1;CD276;TIRAP;IL33;PTGES;TNFRSF12A;SCARB1;CD14;BLNK
pDCs	CLEC4C;CXCR3;GZMB;IL3RA;IRF7;IRF8;LILRA4;PHEX;PLD4;PTCRA
T_cell_co-inhibition	BTLA;C10orf54;CD160;CD244;CD274;CTLA4;HAVCR2;LAG3;LAIR1;TIGIT

T_cell_co-stimulation	CD2;CD226;CD27;CD28;CD40LG;ICOS;SLAMF1;TNFRSF18;TNFRSF25;TNFRSF4;TNFRSF8;TNFRSF9;TNFSF14
T_helper_cells	CD4
Tfh	PDCD1;CXCL13;CXCR5
Th1_cells	IFNG;TBX21;CTLA4;STAT4;CD38;IL12RB2;LTA;CSF2
Th2_cells	PMCH;LAIR2;SMAD2;CXCR6;GATA3;IL26
TIL	ITM2C;CD38;THEMIS2;GLYR1;ICOS;F5;TIGIT;KLRD1;IRF4;PRKCQ;FCRL5;SIRPG;LPXN;IL2RG;CCL5;LCK;TRAF3IP3;CD86;MAL;LILRB1;DOK2;CD6;PAG1;LAX1;PLEK;PIK3CD;SLAMF1;XCL1;GPR171;XCL2;TBX21;CD2;CD53;KLHL6;SLAMF6;CD40;SIT1;TNFRSF4;CD79A;CD247;LCP2;CD3D;CD27;SH2D1A;FYB;ARHGAP30;ACAP1;CST7;CD3G;IL2RB;CD3E;FCRL3;CORO1A;ITK;TCL1A;CYBB;CSF2RB;IKZF1;NCF4;DOCK2;CCR2;PTPRC;PLAC8;NCKAP1L;IL7R;45175;CD28;STAT4;CD8A;LY9;CD48;HCST;PTPRCAP;SASH3;ARHGAP25;LAT;TRAT1;IL10RA;PAX5;CCR7;DOCK11;PARVG;SPNS1;CD52;HCLS1;ARHGAP9;GIMAP6;PRKCB;MS4A1;GPR18;TBC1D10C;GVINP1;P2RY8;EVI2B;VAMP5;KLRK1;SELL;MPEG1;MS4A6A;ARHGAP15;MFNG;GZMK;SELPLG;TARP;GIMAP7;FAM65B;INPP5D;ITGA4;MZB1;GPSM3;STK10;CLEC2D;IL16;NLRC3;GIMAP5;GIMAP4;IFFO1;CFH;PVRIG;CFHR1
Treg	IL12RB2;TMPRSS6;CTSC;LAPTM4B;TFRC;RNF145;NETO2;ADAT2;CHST2;CTLA4;NFE2L3;LIMA1;IL1R2;ICOS;HSDL2;HTATIP2;FKBP1A;TIGIT;CCR8;LTA;SLC35F2;IL21R;AHCYL1;SOCS2;ETV7;BCL2L1;RRAGB;ACSL4;CHRNA6;BATF;LAX1;ADPRH;TNFRSF4;ANKRD10;CD274;CASP1;LY75;NPTN;SSTR3;GRSF1;CSF2RB;TMEM184C;NDFIP2;ZBTB38;ERI1;TRAF3;NAB1;HS3ST3B1;LAYN;JAK1;VDR;LEPROT;GCNT1;PTPRJ;IKZF2;CSF1;ENTPD1;TNFRSF18;METTL7A;KSR1;SSH1;CADM1;IL1R1;ACP5;CHST7;THADA;CD177;NFAT5;ZNF282;MAGEH1
Type_I_IFN_Reponse	DDX4;IFIT1;IFIT2;IFIT3;IRF7;ISG20;MX1;MX2;RSAD2;TNFSF10
Type_II_IFN_Reponse	GPR146;SELP;AHR

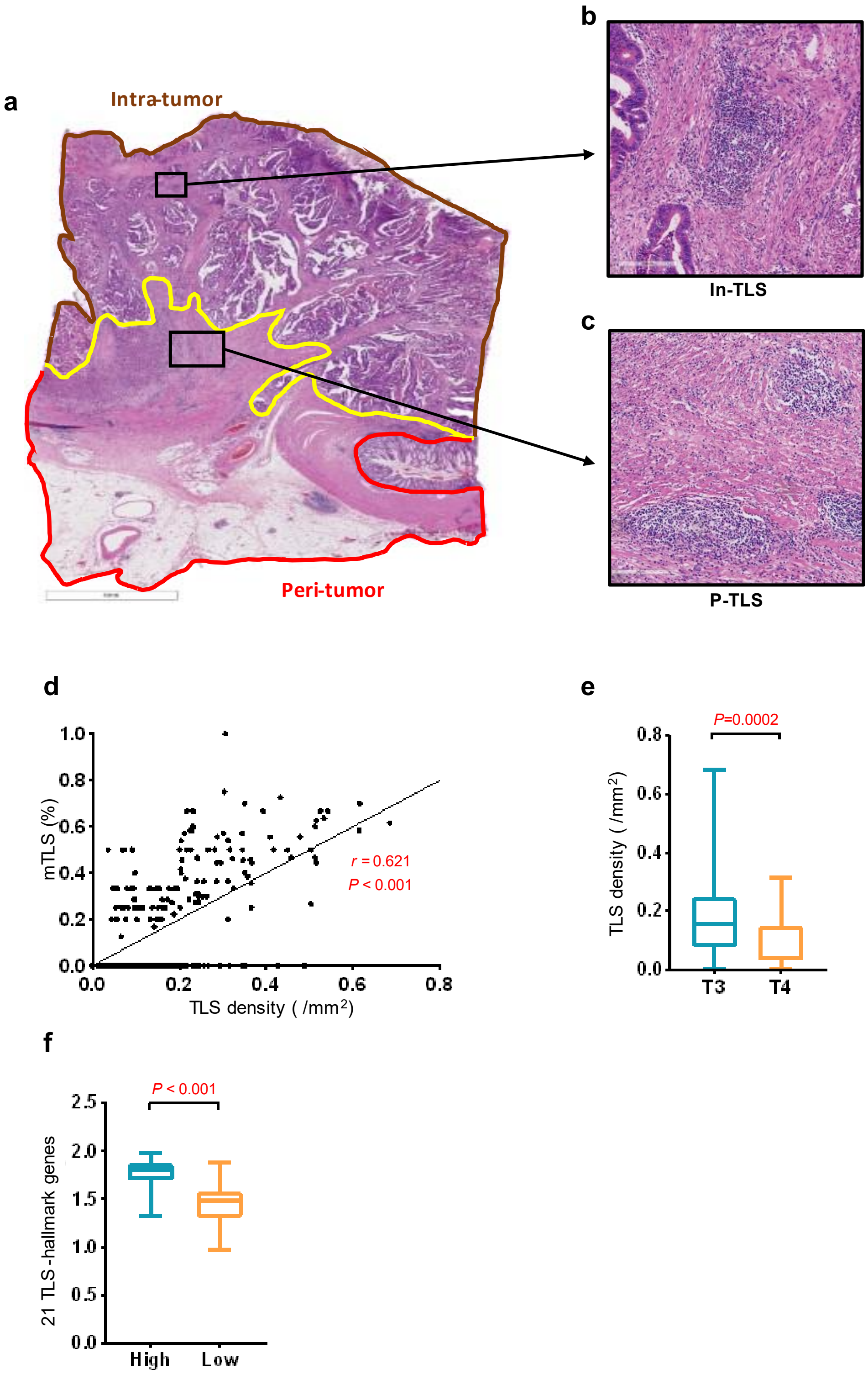

Supplementary Fig. 1 The expression of TLS and its correlates with Clinicopathological characteristics in NT patients. a The peri-tumor region was surrounded in red while the intra-tumor region was surrounded in brown (scale bars, 4 mm). In-TLS (**b**) and P-TLS (**c**) within the peri-tumor region and the intra-tumor region, respectively (scale bars, 200 μm). **d** The correlation between mTLS% and TLS density. **e** TLS density were compared in NT patients with pT4 and pT3. **f** 21 TLS-hallmark genes were compared in patients with TLS-high and –low density patients. In the graphs, the plotted values encompass the range from the minimum to the maximum, with the inclusion of the median.

Supplementary Code:

```
#
library(GSVA)
library(limma)
library(GSEABase)
expFile="symbol.txt"            #
gmtFile="immune.gmt"            #
setwd("C:\\biowolf\\ssGSEA\\12.ssGSEA")            #

#
rt=read.table(expFile, header=T, sep="\t", check.names=F)
rt=as.matrix(rt)
rownames(rt)=rt[,1]
exp=rt[,2:ncol(rt)]
dimnames=list(rownames(exp),colnames(exp))
mat=matrix(as.numeric(as.matrix(exp)),nrow=nrow(exp),dimnames=dimnames)
mat=avereps(mat)
mat=mat[rowMeans(mat)>0,]

#
geneSet=getGmt(gmtFile, geneIdType=SymbolIdentifier())

#ssgsea
ssgseaScore=gsva(mat, geneSet, method='ssgsea', kcdf='Gaussian', abs.ranking=TRUE)
#ssGSEA score
normalize=function(x){
        return((x-min(x))/(max(x)-min(x)))}
#ssGSEA score
ssgseaOut=normalize(ssgseaScore)
ssgseaOut=rbind(id=colnames(ssgseaOut),ssgseaOut)
write.table(ssgseaOut, file="ssgseaOut.txt", sep="\t", quote=F, col.names=F)
```
