# Supplementary material for: Tertiary lymphoid structures predict survival and response to neoadjuvant therapy in locally advanced rectal cancer
Source: NPJ Precis Oncol. 2024 Mar 2;8:61. doi: 10.1038/s41698-024-00533-w (PMC10908779; doi:10.1038/s41698-024-00533-w)
Supplement: Supplementary file 2 — REPORTING SUMMARY [file 41698_2024_533_MOESM2_ESM.pdf]

nature portfolio

Corresponding author(s): DAPR Manuscript # NPJPRECISIONONCOLOGY-04118R1

Last updated by author(s): Jan 24, 2024

Reporting Summary

Nature Portfolio wishes to improve the reproducibility of the work that we publish. This form provides structure for consistency and transparency in reporting. For further information on Nature Portfolio policies, see our Editorial Policies and the Editorial Policy Checklist.

Statistics

For all statistical analyses, confirm that the following items are present in the figure legend, table legend, main text, or Methods section.

n/a	Confirmed	
☐	☒	The exact sample size (*n*) for each experimental group/condition, given as a discrete number and unit of measurement
☒	☐	A statement on whether measurements were taken from distinct samples or whether the same sample was measured repeatedly
☐	☒	The statistical test(s) used AND whether they are one- or two-sided<br>*Only common tests should be described solely by name; describe more complex techniques in the Methods section.*
☐	☒	A description of all covariates tested
☒	☐	A description of any assumptions or corrections, such as tests of normality and adjustment for multiple comparisons
☐	☒	A full description of the statistical parameters including central tendency (e.g. means) or other basic estimates (e.g. regression coefficient) AND variation (e.g. standard deviation) or associated estimates of uncertainty (e.g. confidence intervals)
☐	☒	For null hypothesis testing, the test statistic (e.g. *F*, *t*, *r*) with confidence intervals, effect sizes, degrees of freedom and *P* value noted<br>*Give P values as exact values whenever suitable.*
☒	☐	For Bayesian analysis, information on the choice of priors and Markov chain Monte Carlo settings
☐	☒	For hierarchical and complex designs, identification of the appropriate level for tests and full reporting of outcomes
☒	☐	Estimates of effect sizes (e.g. Cohen's *d*, Pearson's *r*), indicating how they were calculated

Our web collection on statistics for biologists contains articles on many of the points above.

Software and code

Policy information about availability of computer code

Data collection

We used the GSVA package in ssGSEA to determine the immunological characteristics, such as immune cells and immune-related pathways. The statistical analysis software used in this study was R version 4.2.1.

Data analysis

```
library(GSVA)
library(limma)
library(GSEABase)
expFile="symbol.txt"
gmtFile="immune.gmt"
setwd("C:\\biowolf\\ssGSEA\\12.ssGSEA")

rt=read.table(expFile, header=T, sep="\t", check.names=F)
rt=as.matrix(rt)
rownames(rt)=rt[,1]
exp=rt[,2:ncol(rt)]
dimnames=list(rownames(exp),colnames(exp))
mat=matrix(as.numeric(as.matrix(exp)),nrow=nrow(exp),dimnames=dimnames)
mat=avereps(mat)
mat=mat[rowMeans(mat)>0,]

geneSet=getGmt(gmtFile, geneIdType=SymbolIdentifier())
```

```
#ssgsea
ssgseaScore=gsva(mat, geneSet, method='ssgsea', kcdf='Gaussian', abs.ranking=TRUE)
#ssGSEA score
normalize=function(x){
 return((x-min(x))/(max(x)-min(x)))}
#ssGSEA score
ssgseaOut=normalize(ssgseaScore)
ssgseaOut=rbind(id=colnames(ssgseaOut),ssgseaOut)
write.table(ssgseaOut, file="ssgseaOut.txt", sep="\t", quote=F, col.names=F)
```

For manuscripts utilizing custom algorithms or software that are central to the research but not yet described in published literature, software must be made available to editors and reviewers. We strongly encourage code deposition in a community repository (e.g. GitHub). See the Nature Portfolio guidelines for submitting code & software for further information.

Data

Policy information about availability of data

All manuscripts must include a data availability statement. This statement should provide the following information, where applicable:

- Accession codes, unique identifiers, or web links for publicly available datasets
- A description of any restrictions on data availability
- For clinical datasets or third party data, please ensure that the statement adheres to our policy

The datasets used and/or analyzed in the current study presented in the study are included in the article/ Supplementary Material. Further inquiries can be directed to the corresponding author (M.D. Junfeng Du, E-mail: dujunfeng@301hospital.com.cn) based on reasonable written request. The ethics committee and informed consent signed by the participants does not allow for the de-identified RNA sequencing data to be deposited into a secure access-controlled repository.

Research involving human participants, their data, or biological material

Policy information about studies with human participants or human data. See also policy information about sex, gender (identity/presentation), and sexual orientation and race, ethnicity and racism.

Reporting on sex and gender	Our study primarily focuses on the biological attribute of sex, and our findings indicate that there are no significant differences between the sexes in our investigation. We have used the term "sex" carefully, acknowledging it as a biological attribute. Our study design takes into account the consideration of sex, and we explicitly state that our results do not demonstrate any discernible differences between the sexes. While our study does not delve into gender-based analyses, we have ensured that our reporting aligns with your guidelines. We affirm our commitment to ethical practices and have obtained consent for sharing individual-level data, and we are prepared to furnish evidence of this consent as needed.
Reporting on race, ethnicity, or other socially relevant groupings	Our manuscript does not include Reporting on race, ethnicity, or other socially relevant groupings.
Population characteristics	Clinicopathological features included age, sex, tumor location (tumor distance from the anal verge), adjuvant chemotherapy, tumor grade, tumor stage, vascular invasion and perineural invasion. As systemic inflammation markers, the blood-based neutrophil-to-lymphocyte ratio (NLR) was calculated based on pre-operative data.
Recruitment	This study included 242 LARC patients receiving no treatment (NT) at two different institutes, including the 7th Medical Center of Chinese PLA General Hospital and Affiliated Drum Tower Hospital of Nanjing University Medical School between January 2015 to January 2017. All patients underwent curative surgical resection without neoTx, such as chemotherapy (CT) or chemoradiotherapy (CRT). Additionally, this study included a different cohort of 221 neoTx-treated RC patients at the same institutions from January 2013 to January 2018. The pre-neoTx endoscopic biopsies were obtained through transenteroscopic biopsy in neoTx-treated RC patients. Furthermore, 185 residual cancer tissue samples after neoTx were obtained in this neoTx-treated RC patients.
Ethics oversight	The study was approved by the Institute Research Ethics Committees of the 7th Medical Center of Chinese PLA General Hospital (2020-32) and Affiliated Drum Tower Hospital of Nanjing University Medical School (2019-312-01). Written informed consent was obtained from the patient for publication of this case report and accompanying images.

Note that full information on the approval of the study protocol must also be provided in the manuscript.

Field-specific reporting

Please select the one below that is the best fit for your research. If you are not sure, read the appropriate sections before making your selection.

☒ Life sciences ☐ Behavioural & social sciences ☐ Ecological, evolutionary & environmental sciences

For a reference copy of the document with all sections, see nature.com/documents/nr-reporting-summary-flat.pdf

Life sciences study design

All studies must disclose on these points even when the disclosure is negative.

Sample size

This study was a retrospective study, so the larger the sample size, the better. We included 242 LARC patients and 221 patients who received neoadjuvant therapy. Compared with other literature, our sample size is sufficient.

Data exclusions

This study included 242 LARC patients receiving no treatment (NT) at two different institutes, including the 7th Medical Center of Chinese PLA General Hospital and Affiliated Drum Tower Hospital of Nanjing University Medical School between January 2015 to January 2017. All patients underwent curative surgical resection without neoTx, such as chemotherapy (CT) or chemoradiotherapy (CRT).

Replication

In our study, we implemented a series of measures to ensure the reproducibility of experimental findings, particularly focusing on the key techniques of immunohistochemistry (IHC) and mRNA-seq sequencing.
1. Immunohistochemistry (IHC):
Measures: We ensured the reproducibility of IHC experiments by meticulously documenting and standardizing experimental procedures, including tissue processing, antibody selection, and staining protocols.
Results: All attempts at replicating the IHC experiments were successful, indicating the consistent and reliable nature of our experimental procedures.
2. mRNA-seq Sequencing:
Measures: To ensure the reproducibility of mRNA-seq sequencing, we adhered to strict experimental standards and quality control processes, encompassing sample preparation, sequencing instrument calibration, and data processing.
Results: Replication attempts for mRNA-seq were all successful, signifying the consistency of our sequencing data and the reliability of the obtained gene expression information.

In our study, all attempts to replicate experimental results, including both immunohistochemistry and mRNA-seq sequencing, were successful. This underscores the high reproducibility and stability of our experimental methods. No unreproducible or irreproducible results were identified in our research. Our experimental data and findings are deemed reliable and are open for further validation and exploration within the scientific community

Randomization

In our retrospective study, the allocation of samples or participants into experimental groups was solely based on the retrospective categorization of individuals into two groups: high and low based on the density or maturity of TLS (Tertiary Lymphoid Structures). It's important to note that, given the nature of our study design, we did not employ a randomized allocation of participants, as we did not conduct a traditional experimental intervention.

Blinding

Same as above

Reporting for specific materials, systems and methods

We require information from authors about some types of materials, experimental systems and methods used in many studies. Here, indicate whether each material, system or method listed is relevant to your study. If you are not sure if a list item applies to your research, read the appropriate section before selecting a response.

Materials & experimental systems

n/a	Involved in the study	
☐	☒	Antibodies
☒	☐	Eukaryotic cell lines
☒	☐	Palaeontology and archaeology
☒	☐	Animals and other organisms
☒	☐	Clinical data
☒	☐	Dual use research of concern
☒	☐	Plants

Methods

n/a	Involved in the study	
☒	☐	ChIP-seq
☒	☐	Flow cytometry
☒	☐	MRI-based neuroimaging

Antibodies

Antibodies used

Anti-CD3 Mouse 1:150 ZSGB-BIO TA506064
Anti-CD4 Rabbit 1:200 Abcam ab133616
Anti-CD8 Rabbit 1:300 Abcam ab101500
Anti-CD20 Mouse 1:100 Abcam ab9475
Anti-CD21 Mouse 1:100 ZSGB-BIO TA327627
Anti-CD23 Mouse 1:150 ZSGB-BIO TA801554
Anti-CD45RO Mouse 1:1000 Abcam ab23
Anti-CD68 Rabbit 1:4000 Abcam ab213363
Anti-PNAd Rat 1:100 Biolegend 120802

Validation

Anti-CD3 Mouse 1:150 ZSGB-BIO TA506064 human tonsil
Anti-CD4 Rabbit 1:200 Abcam ab133616 human tonsil

Anti-CD8 Rabbit 1:300 Abcam ab101500 human tonsil
Anti-CD20 Mouse 1:100 Abcam ab9475 human tonsil
Anti-CD21 Mouse 1:100 ZSGB-BIO TA327627 human tonsil
Anti-CD23 Mouse 1:150 ZSGB-BIO TA801554 human tonsil
Anti-CD45RO Mouse 1:1000 Abcam ab23 human tonsil
Anti-CD68 Rabbit 1:4000 Abcam ab213363 human tonsil
Anti-PNAd Rat 1:100 Biolegend 120802 human tonsil

Plants

Seed stocks	None
Novel plant genotypes	None
Authentication	None
